# Hyperoxic Treatment Induces Mesenchymal-to-Epithelial Transition in a Rat Adenocarcinoma Model

**DOI:** 10.1371/journal.pone.0006381

**Published:** 2009-07-28

**Authors:** Ingrid Moen, Anne Margrete Øyan, Karl-Henning Kalland, Karl Johan Tronstad, Lars Andreas Akslen, Martha Chekenya, Per Øystein Sakariassen, Rolf Kåre Reed, Linda Elin Birkhaug Stuhr

**Affiliations:** 1 Department of Biomedicine, University of Bergen, Bergen, Norway; 2 The Gade Institute, University of Bergen, Bergen, Norway; 3 Department of Microbiology, Haukeland University Hospital, Bergen, Norway; 4 Department of Pathology, Haukeland University Hospital, Bergen, Norway; Baylor College of Medicine, United States of America

## Abstract

Tumor hypoxia is relevant for tumor growth, metabolism and epithelial-to-mesenchymal transition (EMT). We report that hyperbaric oxygen (HBO) treatment induced mesenchymal-to-epithelial transition (MET) in a dimetyl-α-benzantracene induced mammary rat adenocarcinoma model, and the MET was associated with extensive coordinated gene expression changes and less aggressive tumors. One group of tumor bearing rats was exposed to HBO (2 bar, pO_2_ = 2 bar, 4 exposures à 90 minutes), whereas the control group was housed under normal atmosphere (1 bar, pO_2_ = 0.2 bar). Treatment effects were determined by assessment of tumor growth, tumor vascularisation, tumor cell proliferation, cell death, collagen fibrils and gene expression profile. Tumor growth was significantly reduced (∼16%) after HBO treatment compared to day 1 levels, whereas control tumors increased almost 100% in volume. Significant decreases in tumor cell proliferation, tumor blood vessels and collagen fibrils, together with an increase in cell death, are consistent with tumor growth reduction and tumor stroma influence after hyperoxic treatment. Gene expression profiling showed that HBO induced MET. In conclusion, hyperoxia induced MET with coordinated expression of gene modules involved in cell junctions and attachments together with a shift towards non-tumorigenic metabolism. This leads to more differentiated and less aggressive tumors, and indicates that oxygen *per se* might be an important factor in the “switches” of EMT and MET *in vivo*. HBO treatment also attenuated tumor growth and changed tumor stroma, by targeting the vascular system, having anti-proliferative and pro-apoptotic effects.

## Introduction

Hypoxia is a common feature in tumors and studies have demonstrated that it promotes aggressive tumor behavior, invasiveness and metastatic potential [1], [2]. The disorganized and dysfunctional tumor vasculature is generally attributed to this deficiency in oxygen supply. As hypoxia represents a hallmark of solid tumor growth and metastasis, we aimed to study the effect of enhanced oxygenation (“the flip of the coin”), by using hyperbaric oxygen (HBO) treatment in a rat tumor model. When oxygen is in solution, it can more easily reach tissue areas where blood cells cannot pass, and can also enable tissue oxygenation even with impaired haemoglobin carriage [3]. As in normal tissue, the oxygen partial pressure (pO_2_) in tumor tissue increases significantly during HBO exposure [4] and the pO_2_ elevation lasts for up to 60 min *post* HBO treatment [5]. This type of therapy is frequently used to treat a number of diseases including carbon monoxide poisoning and non-healing wounds [6]. Furthermore, HBO co-treatment has been used extensively and successfully to potentiate therapeutic effects of chemotherapy and radiotherapy, both clinically and in animal models [7]. Recent studies have also concluded that HBO has a significant inhibitory effect *per se* on the growth of mammary tumors [8], [9] and BT4C glioma xenografts in rats [10].

Various cellular functions are influenced by the availability of oxygen, via different molecular sensor-systems regulating signalling, metabolism and gene-expression. The transcriptional complex Hypoxia inducible factor-1 (HIF-1) is a major oxygen sensitive regulator [11]. HIF-1 is regulated through hydroxylation, poly-ubiquitylation and proteasomal degradation of the HIF-1α subunit under normoxic conditions. Under hypoxic conditions HIF-1α is stabilised, and this results in the transcriptional induction of hypoxia-responsive genes encoding proteins that will promote O_2_ delivery (VEGF, erythropoietin) and mediate metabolic adaption to reduced O_2_ availability (glucose transporter-1, glycolytic enzymes) [12]. Through this signalling-pathway, enhanced pO_2_ during and after HBO may influence gene expression and cellular responses, also in tumors.

In recent years, several investigations have suggested that the process of epithelial–to-mesenchymal transition (EMT) may be crucial for carcinogenesis and cancer progression [13], [14], [15], [16], [17]. Cannito *et.al.* [18] provided evidence that tumors exposed to moderate hypoxic conditions can trigger a highly conserved cellular program. EMT is a fundamental process that governs morphogenesis in multicellular organisms [16]. In tumor progression, EMT is involved in a dedifferentiation program that leads to malignant carcinomas with an invasive or metastatic phenotype [15], [16], [19]. Cell morphology, adhesion to other cells and the extracellular matrix (ECM), and migration potential are all features that change during this transition. Loss of E-cadherin (CDH1) expression is one of the hallmarks of EMT, as CDH1 expression is one of the important features of the epithelial phenotype. CDH1 is a cell-cell adhesion molecule that participates in homotypic, calcium-dependent interactions to form epithelial adherens junctions. Several transcription factors have been associated with the repression of CDH1, including zinc finger proteins of the SNAIL and TWIST families, δEF1/ZEB1/TCF8, SIP1/ZEB2/ZFHX1B and the basic helix-loop-helix factor E12/E47 [15]. Loss of CDH1 is also associated with a gain of N-cadherin (CDH2), in a process known as the “cadherin switch”. CDH2 enhances tumor cell motility and migration and has been postulated to exert a dominant effect over CDH1 function. CDH2-dependent motility may be mediated by fibroblast growth factor receptor (FGF-receptor) signalling [20], [21] and this process is potentially implicated in EMT.

Oxygen is a prerequisite for mitochondrial respiration, and an important regulator of energetic pathways. Normal cells utilize glycolysis and mitochondrial oxidative phosphorylation as the two major ATP generating pathways under normoxic conditions. For example in muscle, glycolysis is stimulated under conditions when insufficient oxygen supply limits oxidative phosphorylation, which consequently leads to increased lactate production. Most solid tumors develop a metabolic phenotype that includes high rates of glycolysis and lactate production, even under normoxic conditions [22]. The presence of such “aerobic glycolysis”, combined with low rates of mitochondrial respiration, is now recognized as a general feature of solid tumors (the Warburg effect), and tumor energy metabolism is therefore regarded as a promising therapeutic target [23].

Thus, the aim of the present study was to elucidate the effect of 4 treatments of 2 bar pure oxygen on tumor growth and on factors that influence tumor growth such as angiogenesis, cell death, proliferation, collagen density and metabolism. In addition, we aimed to investigate the influence of hyperoxia on the gene expression profile. The results indicate that oxygen by itself can induce MET in an *in vivo* cancer model.

## Materials and Methods

### Animals and tumor model

A total of 40 female Sprague-Dawley rats (Møllegård, Denmark) were used. Mammary tumors (adenocarcinomas) were induced by dimetyl-α-benzantracene (DMBA) dissolved in olive oil and given to the rat by gavage at the age of 7 weeks at a dose of 16 mg. The experiments were performed when the rats were 13–15 weeks old, having reached a bodyweight of approximately 250 g and developed one to three tumors along the mammary crest. All the experiments were performed in accordance with recommendations of the Norwegian State Commission for Laboratory Animals and experimental procedures were approved by the local ethical committee.

### Hyperbaric chamber

A 30 l pressure chamber (Skjønndal Slipp, Bergen, Norway) with an inner diameter of 25 cm, and an inner length of 65 cm was used. The chamber was supplied with pure O_2_, and the percent of oxygen was monitored continuously by an oxygen meter (NUI, Bergen, Norway). After reaching 100% O_2_ within approximately 10 min, the pressure was raised over a period of approximately 3 min to 2 bar. The 2 bar pure oxygen atmosphere was maintained for a period of 90 min. To maintain>97% O_2_ atmosphere at all times, the chamber was flushed with pure oxygen for 3–5 min every 10–30 min depending on the number of animals in the chamber.

### Experimental groups and treatment design

The HBO treated rats were exposed to 2 bar pure oxygen for 90 min at day 1, 4, 7 and 10, and rats were killed day 11. The controls were exposed to normal atmospheric pressure and air for an equal period of time.

### Measurements of tumor growth

Tumor size was measured externally with a caliper at day 1 (*pre* hyperoxic exposure) and day 11 (*post* hyperoxic exposure) and estimated according to the formula: π/6 ·(a^2^)·(b), where a is the shortest and b is the longest transversal diameter [24]. The rats were anesthetized by isoflurane (Rhone-Poulenc Chemicals, France) and N_2_O and body-temperature was kept at 37±0.5°C.

### Immunohistochemistry

The animals were sacrificed with saturated KCl during anaesthesia and the tumors were immediately dissected out and put into liquid N_2_ and then stored at −80°C or fixed in 4% formalin, processed and embedded in paraffin until use.

Frozen tumor sections (20 µm) were used for immunostaining of blood vessels using mouse anti-rat CD31 (Serotec, MCA 1334G, Düsseldorf, Germany) as primary antibody and biotinylated rabbit anti-mouse (DAKO, no E0413, Oslo, Norway) as secondary antibody. Diaminobenzidine (DAB) was used as a chromogen to visualize blood vessels, and hematoxylin (H & E, Merck, Damstadt, Germany) was used a nuclear counterstain. The cross-sectional density of CD31 positive structures was quantified per µm^2^ using a counter grid, while the blood vessel diameter was measured on longitudinal structures in “hotspot” areas from 10 (40×) high power fields (HPF) for each tumor. Staining for CDH2 was performed on frozen sections of tumor tissue using the monoclonal mouse CDH2 antibody M3613 (DAKO, Copenhagen, Denmark) diluted 1∶25 and incubated for 1 hr at room temperature. Staining for CDH1 was performed on frozen sections of tumor tissue using the rabbit polyclonal antibody Ab53226 (Abcam, Cambridge, UK) diluted 1∶50 and incubated for 2 hrs at room temperature. Staining was done using the EnVision-labeled polymer method, with commercial kits (DAKO). The antigens were localized by the diaminobenzidine tetrachloride peroxidase reaction, and the slides were counterstained with Mayer's hematoxylin.

Paraffin embedded tumor sections (10 µm) were used for proliferation and apoptosis staining. Tumor cell proliferation was assessed by staining with an anti-rat Ki67 monoclonal antibody diluted 1∶15 (No. M7248, Dako Cytomation, Denmark), and secondary antibody from DAKO envision kit (DACO Patts, Glostrup, Denmark). Cell death was examined by the terminal transferase-mediated dUTP nick-end-labeling (TUNEL) method (Boehringer Mannheim, Mannheim, Germany), performed according to the manufacturers recommendation, as described in reference [10]. Tumor proliferation was quantified as the percent of Ki67 positive cells per visionfield. All sections were examined using a Nikon light microscope (THP Eclipse E600, Nikon Corporation, Tokyo, Japan) and the images were captured with a Nikon Digital Camera (DXM 1200F, Nikon Corporation, Tokyo, Japan).

### Determination of collagen content in the tumors

Transmission electron microscopy (TEM) was used to examine tumor collagen fibrils. The tumor pieces were fixed in 2.5% glutaraldehyde in 0.2M (CH3)^2^ As( = o)ONa and stained with osmium using standard procedures. The pieces were then moulded into Agar 100 Resin plastic and sliced into 60 nm thick sections, before they were marked with lead-citrate and uranyl-acetate and analysed in a transmission electron microscope (JEM-1230, Jeol, Japan). The number of collagen fibrils on representative cross-sectional pictures each of 29 µm^2^ from both control and repeated HBO treated tumors were counted, and the average number of fibrils per µm^2^ were calculated.

### Global Gene Expression Analysis

Global gene expression analysis was performed, studying changes in gene clusters, in order to identify key molecular mechanisms and changes in gene programs. Changes in gene-expression profiles after HBO suggested several relevant pathways to be involved. A total of 6 controls and 4 HBO treated tumors were analysed. The Agilent G413F Whole Rat Genome (4×44k) Oligo Microarray Kit with SurePrint Technology (Agilent Technologies, Inc., Palo Alto, CA) was used to analyze samples in the present study. One µg of DNAse-treated total RNA was converted into cDNA and Cy3-labeled cRNA using the Low RNA Input Linear Amplification Kit PLUS, One-Color kit (Agilent Tech., Santa Clara, CA, USA) as previously described [17]. The normalized channel values were log(2) transformed and combined into a gene expression data matrix. Data were formatted in a J-Express-file suitable for additional data mining (http://www.molmine.com/) [25]. Following normalization, the tumor samples were divided in two groups, the HBO treated and non-treated tumors. We used analysis of variance (ANOVA), t-score analysis and SAM (Significant Analysis of Microarray) of the J-Express program package for identification of differentially expressed genes. Annotated microarray data were uploaded in the BASE database and formatted and exported to ArrayExpress at the European Bioinformatics Institute (http://www.ebi.ac.uk/microarray/) in agreement with the MIAME guidelines (E-TABM-718).

### Statistics

Unpaired t-test was used to compare results between the groups (angiogenesis, proliferation, apoptosis, and tumor growth). Paired t-test was used when comparing results within the same group of animals. A value of p<0.05 was considered statistically significant. SigmaPlot 8.0 (Alfasoft AS, Lillestrøm, Norway) was used for the statistical evaluation.

## Results

### Tumor volume

**Figure 1:**
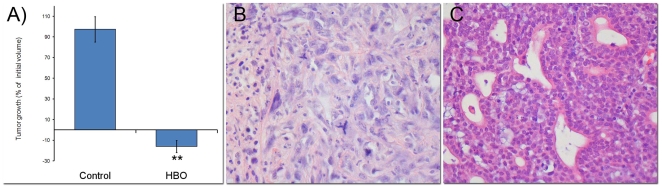
Tumor growth and morphology. Tumor growth (% of initial volume) in control and hyperbaric oxygen (HBO) treated tumors after 11 days (A). Box plot presented as means ± SEM. ** p<0.001 compared to controls. DMBA-induced mammary control tumors showed mostly poorly differentiated growth with high cellularity and marked nuclear pleomorphism (B). After HBO treatment, a majority of the cases appeared to have a more differentiated morphology, with glandular structures and less marked cellular atypia and showed large areas of cell death (C).

A total of 20 controls and 20 HBO treated tumors were measured. The measurements of tumor volume started approximately 5 weeks post DMBA induction, when the tumors were approximately 1.0–2.5 cm^3^. During the observation period of 11 days, tumor volume increased significantly in controls (p<0.0001), whereas a marked reduction in tumor volume (p<0.0001) was found in HBO treated tumors compared to pre-treatment sizes at day 1 (Fig. 1A).

### Hyperoxia changes tumor morphology and cadherin expression

**Figure 2:**
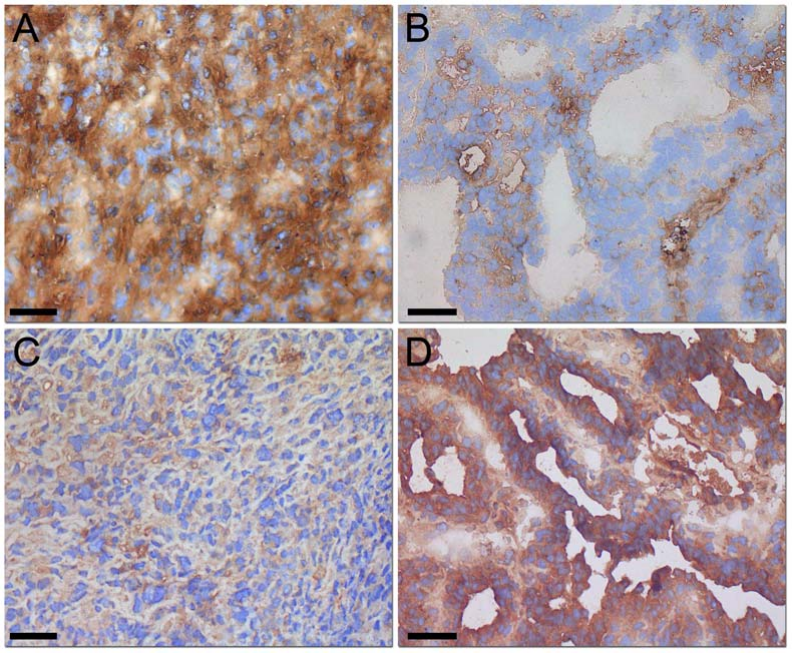
Cadherin expression (CDH1 and CDH2). Expression of CDH2 protein (N-cadherin) by immunohistochemistry was weaker in the HBO treated, differentiated tumors (B) and was found to be clearly increased in the less differentiated control tumors (A), whereas the opposite was found for CDH1 (E-cadherin) expression (C and D; ×400).

DMBA-induced mammary control tumors showed mostly undifferentiated growth with high cellularity and marked nuclear pleomorphism (n = 6) (Fig. 1B). However, after HBO treatment a majority of the cases appeared to have a more differentiated morphology, with glandular structures and less marked cellular atypia as well as areas of cell death (n = 5) (Fig. 1C). Expression of CDH2 protein was weaker in the HBO treated, differentiated tumors and was found to be clearly increased in the less differentiated control tumors, whereas the opposite was found for CDH1 expression (Fig. 2). In positive cases, staining was uniform with distinct protein expression in a majority of the tumor cells.

### Hyperoxia changes tumor vascular density and diameter

To determine the impact of hyperoxia on the tumor vasculature, the vascular density in “hot spot” areas both in the tumor centre and periphery, was examined. The number of CD31 positive tumor blood vessels per mm^2^ were significantly reduced (∼40%) after HBO treatment (n = 5 for both groups), as shown in Table 1. The tumor vessel diameter was also changed after HBO. A reduction in vessel diameter was found in the periphery of the tumor, while centrally the vessels were dilated (Table 1). Microarray analysis confirms the anti-angiogenic effect of HBO on DMBA-induced tumors, with reduced levels of growth factors like *Vegf, Fgf and Tgf* (Table 2).

**Table 1:** Proliferation, blood vessel density and apoptosis in controls and after hyperbaric oxygen exposure (HBO). (Mean values ± SEM).

	Control	HBO
**Blood vessel density** (numbers/mm2)		
Centrally	43.6±7.7	17.4±7.9**
Periferally	39.2±5.7	15.7±6.0***
**Blood vessel diameter** (µm^2^)		
Centrally	57±3.2	77.3±7.2***
Periferally	58.9±3.4	30.3±2.6***
**Proliferation** (% Ki67 positive tumor cells)	19±2.9	9±1.0 **
**Apoptosis** (% of total area)	8.6±3.3	34.7±5.6***

** p<0.02 vs control.

*** p<0.001 vs control.

### Hyperoxia reduces tumor cell proliferation

The effect of hyperoxia on tumor growth may be due to a reduction in tumor cell proliferation. We therefore immunostained the tumors for the cell proliferation marker Ki67, and interestingly, the proportion of Ki67 positive tumor cells were less in the HBO exposed group (n = 5 for both groups). Thus, hyperoxia induces a significant reduction in proliferating tumor cells (Table 1). Gene expression profile analysis confirms the regulation of tumor growth factors (Table 2).

**Table 2:** Markers of apoptosis, tumor growth, fibrosis and metabolism in HBO treated mammary adenocarcinoma.

Genes	Gene name	Expression	p-values
**Apoptosis facilitators**			
*Perp*	TP53 apoptosis effector	60	5.2E-21
*Bik*	Bcl2-interacting killer	5.7	5.1E-08
*Casp6*	Caspase 6	4.1	1.3E-05
*Bbc3*	PUMA	2.0	4.1E-05
*Bak1*	BCL2-antagonist/killer 1	2.0	2.1E-05
*Bid3*	BH3 interacting domain death agonist	1.5	1.3E-02
*Bax*	Bcl-associated X protein (apoptosis-inducing)	1.5	7.2E-04
*Bnip3l*	BCL2/adenovirus E1B 19 kDa-interacting protein 3	1.3	3.0E-04
**Apoptosis inhibitors**			
*Bcl2l2*	Bcl2-like2	1.8	1.7E-7
*Bcl2*	Bcl-2 beta protein	1.3	4.1E-3
*Birc3*	Baculoviral IAP repeat-containing 3	0.8	2.6E-3
*Ciapin1*	Cytokine induced apoptosis inhibitor1	0.7	2.1E-3
*Becn1*	Autophagy related Beclin 1	0.7	3.6E-4
*Bag1*	Bcl2-associated athanogene 1	0.7	9.9E-4
*Tmbim1*	BAX inhibitor motif containing1	0.3	2.1E-4
*Birc5*	Survivin	0.2	1.1E-4
*Nol3*	Apoptosis repressor with CARD domain	0.2	2.1E-10
*Birc2*	Baculoviral IAP repeat-containing 2	0.1	4.4E-14
**Tumor growth factors**			
*Vegfα*	Vascular endothelial growth factor A	0.1	9.8E-9
*Vegfβ*	Vascular endothelial growth factor B	0.3	1.7E-7
*Fgfr1*	Fibroblast growth factor receptor 1	0.2	1.1E-7
*Tgfά*	Transforming growth factor, alpha	0.3	4.1E-7
*Tgfβ1*	Transforming growth factor, beta 1	0.6	1.9E-4
*Tgfβ2*	Transforming growth factor, beta 2	0.3	2.3E-5
*Tgfβr1*	Transforming growth factor, beta receptor 1	0.6	1.3E-4
*Tgfβr2*	Transforming growth factor, beta receptor 2	0.3	2.5E-7
**Fibrosis**			
*Agc1*	Aggrecan 1	0.5	4.1E-4
*Lum*	Lumican	0.5	8.8E-3
*Fmod*	Fibromodulin	0.2	1.5E-3
**Metabolism**			
*Ldhb*	Lactate dehydrogenase B	10	1.0E-5
*Gapdh*	Glyceraldehyde-3-phosphate dehydrogenase	0.5	1.3E-4
*Ldha*	Lactate dehydrogenase A	0.4	1.3E-8
*Hk2*	Hexokinase II	0.2	1.6E-7

Displayed results are fold change of gene expression in HBO treated rat mammary adenocarcinomas compared to untreated tumors.

### Hyperoxia enhances cell death

To determine whether the reduction in tumor growth was related to induction of cell death, we performed TUNEL staining, and counted the TUNEL labeled cells in the tumors (n = 5 for both groups). As shown in Table 1, the number of dead cells in the tumor was significantly increased (p<0.001) after HBO treatment. Genes related to apoptosis are elucidated in Table 2, confirming the immunohistochemical finding.

### Hyperoxia influences fibrosis

**Figure 3:**
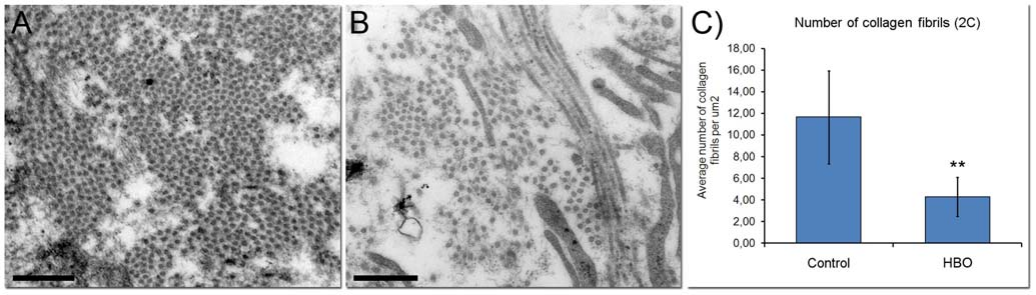
Collagen density. The collagen fibril density is shown in control (A) and hyperbaric oxygen (HBO) treated tumors (B) on cross-section fibrils magnified 50.000 times. Scale bar indicates 0.5 µm. Manually counted collagen fibril density ± SEM (C). **p<0.001 compared to controls.

There were pronounced differences in collagen fibril density in tumor stroma between control (n = 5) and HBO treated (n = 4) tumors, as displayed in Figures 3A and B. The manually counted fibril density showed a statistically significant decline in fibril density after HBO treatment (Fig. 3C) (p<0.001). The results are supported by microarray analysis, showing down-regulation of several fibrosis-related genes (Table 2).

### Hyperoxia induces mesenchymal-to-epithelial transition

Whole genome gene expression profiling was used to investigate if hyperoxia could influence EMT in an *in vivo* tumor model. Table 3 presents expression values of epithelial and mesenchymal cell markers in HBO treated tumors compared to untreated control tumors. The results show a significant shift from mesenchymal to epithelial cell marker expression. A significant up-regulation of most of the genes involved in maintenance of the epithelial phenotype, like *Cdh1*, and a significant down-regulation of *Cdh2*, a marker of the mesenchymal phenotype, was found. *Cdh1* inhibitors such as *Snai1* and *Twist2* were significantly down-regulated in the HBO treated tumors compared to control, and could promote the mesenchymal-to-epithelial transition. Furthermore, gene expression of entire cell junction and attachment modules such as adherens junctions, gap junctions, tight junctions, desmosomes and hemidesmosomes were coordinately and strongly induced in the HBO treated rats (Supplementary Table S1). Selected gene expressions were validated by immunohistochemical staining (Fig. 2).

**Table 3:** Mesenchymal-to-epithelial transition markers in HBO treated mammary adenocarcinoma.

Genes	Expression	p-values
**Epithelial markers**		
*Areg*	280	1.6E-11
*Krt14*	173	1.5E-12
*Krt5*	138	8.7E-14
*Cdh1*	88	1.9E-6
*Perp*	60	5.2E-21
*Dsp*	25	8.4E-14
*Serpinb*	10	1.2E-5
*Ocln*	7	6.0E-10
*Cdh3*	1.3	2.8E-3
**Mesenchymal markers**		
*Snai1*	0.11	2.2E-10
*Fgfr1*	0.17	1.1E-7
*Cdh2*	0.19	4.9E-7
*Fn1*	0.27	5.6E-6
*Cdh11*	0.27	2.1E-3
*S100a4*	0.28	2.2E-11
*Bmp7*	0.28	4.4E-5
*Fzd2*	0.34	2.8E-4
*Twist2*	0.44	3.4E-4
*Nid1*	0.58	2.5E-3
*Vim*	0.67	5.0E-3

Displayed results are fold change of gene expression in HBO treated mammary adenocarcinomas compared to untreated tumors detected by use of Agilent Human Whole Genome Oligo Microarrays and SAM software analysis. The p-values are based upon t-score analysis and SAM and FDR less than 1%.

### Hyperoxia induces a metabolic shift

There were strong indications that the tumor energy metabolism was shifted away from aerobic glycolysis after HBO treatment (Table 2). The glycolytic enzymes hexokinase II and glyceraldehyde 3-phosphate dehydrogenase (*Gapdh*) were significantly reduced after HBO treatment, opposing the tumor specific metabolic glycolytic phenotype. Furthermore, the lactate dehydrogenase (*Ldh*) expression profile was also changed. Our data showed a significant reduction in *Ldha* expression together with a dramatic increase in *Ldhb* expression. The relative changes in *Ldha* and *Ldhb* expression strongly suggest the formation of *Ldh* isoforms with a preference for catalysis in the lactate-pyruvate direction.

## Discussion

The present study showed a significant reduction (∼16%) in DMBA-induced mammary tumor size after 2 bar HBO treatment compared to day 1. This corresponds to the reduction found by Stuhr *et al.* [9] on an identical tumor model and after identical treatment. Raa *et al.* [8] also showed reduction of DMBA-induced tumor growth after normobaric (1 bar) and hyperbaric (1.5 bar) hyperoxia (100% O_2_), although not to the same extent. Taken together, the results indicate that the reduction in DMBA-induced tumor growth is dose-dependent on pO_2_.

Angiogenesis is essential for tumor growth and has been shown to be hypoxia-induced. One might therefore expect that hyperoxia may inhibit the angiogenic switch. However, hyperoxia is known to increase vessel development in normal tissue and in wound healing [26]. Interestingly, we found a significant reduction in mean vascular density after HBO treatment in the present mammary tumors. This indicates that hyperoxia has an anti-angiogenic effect on these tumors. This anti-angiogenic effect applies both for the central and peripheral parts of the tumor. The gene expression analysis supports this, and shows a significant down-regulation of pro-angiogenic genes such as *Vegfα, Vegfβ*, *Fgf, Pdgf* and *Tgf*α after HBO treatment (Table 2). As blood vessels are necessary to nurture the tumor tissue, this anti-angiogenic effect could help explain the restriction in tumor growth. A similar anti-angiogenic effect was found previously in transplanted gliomas after 2 bar pure oxygen treatment [10]. Thus, hyperoxia could potentially be used for anti-angiogenic therapy in tumors.

The mean vascular diameter is also changed after treatment. Peripherally, the vessels are constricted, the same reaction as in normal blood vessels when treated with HBO. In contrast, vessels in the central parts of the tumors are dilated. Raa *et.al.* [8] identified the same dilatory effect in DMBA induced tumors treated with normobaric and hyperbaric (1.5 bar) hyperoxia. We might therefore speculate that this dilation is the tumor's response to elevate its flow, and thereby compensate for loss of blood vessels.

HBO also significantly decreased tumor cell proliferation. This is a direct inhibition of dividing cells, and thereby of tumor growth. A study by Granowitz *et.al.* [27] corresponds to our results by showing that HBO inhibited both benign and malignant human mammary epithelial cell proliferation *in vitro*. They also showed that the anti-proliferative effect of HBO alone was similar in magnitude to the effect of high-dose of melphala, gemcitabine and paclitaxel alone, indicating that HBO could be an effective therapy for breast cancer.

A significant increase in cell death after repeated HBO can also help explain the tumor inhibitory effect. Several factors in the apoptotic machinery are influenced by HBO treatment. The induction of apoptosis by p53 is fundamental and the targets PUMA, BAX, BAK, BIK and PERP play cell type-specific roles in p53-mediated apoptosis. Gene expression analysis shows a strong induction of *Perp*, an apoptotic effector that promotes mitochondrial membrane permeability and is involved in cell adhesion and cell junction as well [28], [29]. The striking picture of increased apoptotic facilitators and down-regulation of apoptotic inhibitors (Table 2) supports the observation of elevated number of TUNEL positive tumor cells after HBO treatment. This also corresponds with previous findings in DMBA-induced mammary tumors after 1 and 1.5 bar HBO treatment [8] and in transplanted gliomas in nude rats after both 1 and 2 bar HBO treatment [10].

Several reports have previously suggested that EMT is crucial for cancer progression [15], [16]. This process converts adherent epithelial cells to mobile mesenchymal appearing cells. Cell-junctions, especially adherens junctions, tight junctions and desmosomes, are required for the epithelial phenotype and for epithelial cells to function as a tissue [30]. A strong induction of genes associated with these cellular junctions was found after HBO treatment indicating a transition towards an epithelial phenotype (Table 3; Supplementary Table S1). The functional loss of CDH1, is a key event in the EMT process [13]. Cowin *et.al.* [20] provided evidence that CDH1 serves both as a tumor suppressor and an invasion suppressor in an animal model of invasive lobular breast cancer (ILC). Several transcription repressors, such as SNAI1, SNAI2 and TWIST have been identified as strong repressors of CDH1 [31], [32]. Our gene expression analysis on DMBA-induced mammary tumors showed that mesenchymal cell markers are significantly down-regulated in 2 bar HBO treated tumors compared to controls. The results show a significant up-regulation of *Cdh1*, concomitant with a down-regulation of *Snai1* and *Twist2* among others (Table 3, Fig. 2). Reexpression of *Cdh1* might counteract migration and metastasis of cancer cells [20]. Reduction of CDH1 is often accompanied by reciprocally increased expression of CDH2 [21], and indeed CDH2 has been shown to promote breast cancer cell invasiveness in various studies [21], [33]. CDH2 is a well regarded marker of EMT and we found a significant down-regulation of *cdh2* (Table 3, Fig. 2), thus confirming MET [19]. The TGF*β* family of cytokines has been shown to promote EMT during cancer progression [31], [34], [35] and this is in accordance with the present study showing a strong down-regulation of *Tgfβ1, Tgfβ2* and the receptors *Tgfβr1* and *2* (Table 2) after HBO treatment. Furthermore, the hedgehog signal pathway is known to promote EMT and cell migration. In the HBO treated adenocarcinomas the many components of the hedgehog pathway were significantly suppressed (data not shown). Thus, the restoration of a MET programme should efficiently slow the dedifferentiation and dissemination of tumor cells, and thereby result in a less aggressive tumor phenotype [16], [36] as we see in Figure 1C. The present study implicates oxygen *per se* as a significant mediator of the MET “switch”. To the best of our knowledge, this is the first study to discover that oxygen per se might be a significant contributor to mesenchymal-to-epithelial transition *in vivo*.

EMT has also shown an important role in fibrosis of lungs, liver, kidney and cancer, and TGF-β is a key mediator of EMT in fibrosis [13], [37]. Tumor stroma is characterized by activated connective tissue cells producing a collagen-rich matrix. It is therefore very interesting that the HBO treated tumors had a reduction in *Tgfβ* (Table 2) and also a concomitant reduction in the number of collagen fibrils (Fig. 3). Additionally, Oldberg *et.al.* [38] described the effect of the small leucine-rich repeat proteoglycan (SLRP), fibromodulin, in collagen assembly and maintenance, producing a dense stroma. They showed that fibromodulin deficiency resulted in altered tissue organization with fewer and abnormal collagen fibril bundles. In accordance with the reduction in fibrosis, our analysis showed a significant down-regulation of fibromodulin in the HBO treated tumors. Additionally, two other fibrosis-related genes, aggrecan1 (*Agc1*) and lumican (*Lum*) are down-regulated in the HBO treated tumors (Table 2). This indicates that hyperoxic induced MET and has an anti-fibrotic effect as well in the present mammary adenocarcinomas.

The energy metabolism in tumors is highly glycolytic, inducing excessive lactate secretion and acidification of the tumor environment which facilitates tumor invasion. Therefore, targeting the energy metabolism has been proposed to represent a possible therapeutic strategy in the treatment of human cancer [23]. The first critical step in glycolysis is phosphorylation of glucose, which is performed by hexokinases. These enzymes participate in glucose catabolism. The present study shows a clear down-regulation of hexokinase II after HBO treatment. Interestingly, we also see a change in the *Ldh* expression profile after HBO treatment, with reduced lactate dehydrogenase A (*Ldha*) and up-regulation of lactate dehydrogenase B (*Ldhb*). The relative abundance of the subunits *Ldha* and *Ldhb* seems to determine direction of the catalytic activity [39]. A higher portion of *Ldhb* leads to increased amounts of pyruvate (from lactate), which is a common substrate for mitochondrial oxidation (Table 2). Taken together these findings indicate a shift towards non-tumorigenic metabolism, and thereby a shift towards tumor- suppressing alterations in signal transduction.

In conclusion, we have shown that hyperoxia induces a coordinated alteration of entire gene modules of cell junctions and attachments and a MET. This leads to more differentiated and less aggressive DMBA-induced mammary tumors, and indicates that oxygen *per se* might be an important factor in the “switches” of EMT and MET *in vivo*. HBO treatment also attenuates tumor growth and changes tumor stroma by targeting the vascular system, having anti-proliferative and pro-apoptotic effects. In addition, hyperoxia shifted the metabolism from glycolysis to oxidative phosphorylation. Thus, as hypoxia acts as a tumor promoter, hyperoxia acts as a tumor suppressor in DMBA induced mammary tumors in rats. However, further studies are needed to elucidate the underlying mechanisms.

## Supporting Information

Table S1 Expression of cell junction genes in HBO treated rat mammary adenocarcinomas compare to control.
(0.08 MB DOC)
