# Supplementary material for: Hyperoxic Treatment Induces Mesenchymal-to-Epithelial Transition in a Rat Adenocarcinoma Model
Source: PLoS One. 2009 Jul 28;4(7):e6381. doi: 10.1371/journal.pone.0006381 (PMC2712688; doi:10.1371/journal.pone.0006381)
Supplement: Table S1 — Expression of cell junction genes in HBO treated rat mammary adenocarcinomas compare to control. (0.08 MB DOC) [file pone.0006381.s001.doc]

|  |
| --- |

**Supplementary Table S1. Expression of cell junction genes in HBO**

**treated rat mammary adenocarcinomas compare to control**

| **Genes** | **Expression** | **p-value** |
| --- | --- | --- |
| ***Desmosomes*** |  |  |
| Plakophilin 3, *Pkp3* | 13 | 1.4E-9 |
| Desmocollin 2, *Dsc2* | 10 | 7.7E-11 |
| Plakophilin 2, *Pkp2* | 10 | 5.1E-6 |
| Plakophilin 4, *Pkp4* | 4 | 2.2E-7 |
|  |  |  |
| ***Adherens junction*** |  |  |
| Cadherin 1, *cdh1* | 88 | 1.9E-15 |
| Catenin alpha-like 1, *Ctnnal1* | 8 | 3.0E-10 |
| Catenin alpha 1, *Ctnnd1* | 2.3 | 7.5E-9 |
| Nectin, *Pvrl1* | 2.3 | 7.7E-13 |
| Cadherin 3, *cdh3* | 1.3 | 2.8E-3 |
| Cadherin 2, *cdh2* | 0.2 | 4.9E-7 |
|  |  |  |
| ***Gap junction*** |  |  |
| Gap junction protein beta2, *Gjb2* | 19 | 4.8E-8 |
| Gap junction protein beta3, *Gjb3* | 6 | 1.6E-9 |
| Gap junction protein beta4, *Gjb4* | 3.5 | 4.4E-6 |
| Gap junction protein beta5, *Gjb5* | 2 | 5.8E-10 |
|  |  |  |
| ***Tight junction*** |  |  |
| Claudin 7, *Cldn7* | 82 | 1.3E-13 |
| Junction adhes molecule 1, *Jam1* | 29 | 3.6E-7 |
| Claudin 3, *Cldn3* | 23 | 12.8E-13 |
| Claudin 4, *Cldn4* | 14 | 6.6E-9 |
| Tight junction protein 3, *Tjp3* | 10 | 6.0E-14 |
| Claudin 1, *Cldn1* | 3 | 5.6E-5 |
| Claudin 10, *Cldn10* | 3 | 7.4E-4 |
| Junction adhes molecule 3, *Jam 3* | 3.5 | 7.0E-6 |
|  |  |  |
| ***Hemidesmosomes*** |  |  |
| Keratin 19, *Krt19* | 520 | 1.1E-13 |
| Keratin 18, *Krt18* | 497 | 4.7E-15 |
| Keratin 14, *Krt14* | 173 | 1.5E-12 |
| Keratin 5, *Krt5* | 138 | 8.7E-14 |
| Keratin 8, *Krt8* | 13 | 2.1E-5 |
| Integrin beta 4, *Itgb4* | 3.8 | 1.0E-8 |
| Integrin alpha 6, *Itga6* | 2 | 1.4E-4 |
|  |  |  |
| ***Focal adhesion*** |  |  |
| Integrin beta 6, *Itgb6* | 156 | 1.6E-12 |
| Laminin, alpha 1, *Lama1_predicted* | 8 | 2.3E-8 |
| Integrin beta 4, *Itgb4* | 3.8 | 1.0E-8 |
| Tensin 4, *Tns4* | 3 | 1.4E-3 |
| Sorbin, *LOC686098* | 3 | 9.7E-6 |
| Laminin, alpha 4, *Lama4* | 2.9 | 1.5E-5 |
| Caveolin 2, *Cav2* | 2 | 4.0E-5 |
| Integrin alpha 6, *Itga6* | 2 | 1.1E-3 |

____________________________________________________________

Expression of cell attachment genes according to Agilent Human Whole

Genome Oligo Microarrays. Displayed results are fold change of gene

expression in HBO treated mammary adenocarcinomas.
